# Congenital abnormality effect of methamphetamine on histological, cellular and chromosomal defects in fetal mice

**Published:** 2013-01

**Authors:** Tahereh Mirjalili, Seyed Mehdi Kalantar, Maryam Shams Lahijani, Mohamad Hasan Sheikhha, Alireza Talebi

**Affiliations:** 1 *Developmental Biology, Animal Sciences, Faculty of Biological Sciences, Shahid Beheshti University, G.C., Tehran, Iran.*; 2 *Department of Medical Genetics, Research and Clinical Center for Infertility, Shahid Sadoughi University of Medical Sciences, Yazd, Iran.*; 3 *Department of Andrology, Research and Clinical Center for Infertility, Shahid Sadoughi University of Medical Sciences, Yazd, Iran.*

**Keywords:** *Methamphetamine*, *Apoptosis*, *Brain*, *Histology*, *Karyotype*, *Abnormality*, *Fetal*

## Abstract

**Background:** Methamphetamine (MA) is a potent psychomotor stimulant with high abuse and addictive potential. MA is a neurotoxic drug which is widely abused by females of childbearing age, raising serious public health concerns in terms of exposure of the fetus to the drug. Neurotoxic effects of MA on adult are well known, such as dopaminergic nerve terminal degeneration and cell death in regions of brain in some doses.

**Objective:** In the present study, we examined effect of prenatal MA exposure on mouse fetuses.

**Materials and Methods:** In this study, forty 8-12 week-old NMRI female mice were used which were mated with male mice in serial days. When sperm plug was observed it was designated as gestational day (GD) 0. Pregnant mice were individually housed in plastic cages. Pregnant mice were divided into four groups: in first group 10 mg/kg /day MA, in second group 5 mg/kg /day MA and in third group saline were injected subcutaneously from GD 6 to GD 14, corresponding to organogenesis period, while fourth or control group were without injection. On GD 14 fetuses were removed and accomplished chromosome preparation from fetal liver. Then fetal were fixed in formalin for brain hematoxilin and eosine staining and TUNEL assay.

**Results:** We observed morphological abnormality including exencephal fetus in 5mg/kg MA group and premature fetuses in 10 mg/kg MA group. Also brain histological study showed subarachnoid hemorrhage in fetal brain in both experimental groups. Fetal liver karyotyping analysis was normal in fetuses of all groups and TUNEL assay in fetal striatum did not show significant difference in number of apoptotic cells between groups.

**Conclusion:** From our results, it could be concluded that chronic abuse of MA by pregnant females during organogenesis period can cause teratogenic effect and brain hemorrage in fetus.

## Introduction

The rapid increase in illicit production and abuse of methamphetamine (MA), particularly this fact that this agent is being abused by women of childbearing age, has become a public health problem (1). Beside treatment and withdrawal of MA addiction is very difficult (2). In spite of MA abuse by pregnant women, relatively the effect of prenatal MA exposure is not well known (3). 

MA abuse by adult human and also in several mammalian species by using various drug administration schedules causes neurodegeneration, such as dopaminergic nerve terminal degeneration, loss of dopamine uptake sites, decreases in dopamine levels, impairment in dopamine transporter vesicular dopamine uptake system, ionic antiporters at the dopaminergic neurons, and decrease in tyrosine hydroxylase activity in the striatum (4-9). Also, high doses of MA treatments cause decrease of brain serotonin levels, and reduction in the number of serotonin transporter binding sites (10, 11). MA abuse causes cell death in various brain regions, including neuronal cell death in hippocampal remnants, enkephalin-positive cells in the striatum, glutamate-positive neurons and astrogliosis in the somatosensory cortex (12-14). 

Results of neurodegeneration are motor coordination impairment relating to the decreased dopamine levels resulting from the dopaminergic neurodegeneration in the striatum**, **slowed fine and gross motor skills relating to decreased striatal dopamine transporters, decreased everyday functioning ability relating to frontal cortex dysfunction, impaired verbal learning, and increased emotional and cognitive problems (2, 15-17). 

Also performed studies by Williams *et al* have shown injections of MA during post-natal days (P) 11-20 in rats, but not from P1 to P10, lead to memory impairment in the water maze and in sensorimotor gating ability (pre-pulse inhibition (PPI), and spatial learning impairment (18). In addition, MA abusers showed risk increase of cerebral vascular accidents, even in young people, such as ischemic stroke, and subarachnoid hemorrhage (19-22). Also MA abuse significantly increases viral load in the brain, but not in the plasma.

In the present study histological changes of mouse fetal brain which exposed to MA *in utero* during organogenesis clinical period was evaluated. Also cell death was examined in striatum and chromosomal changes in mouse fetuses.

## Materials and methods

**Methamphetamine**

MA was provided by Yazd center of combating against drugs and purity of drug was confirmed by using gas chromatography-mass spectrometry (GC/MS) in chemistry laboratory of Tehran Identifying Center. The approval letter was obtained from Research and Clinical Centre for Infertility ethic committee.

**Animals and methamphetamine treatment**

8-12 week-old NMRI mice in animal house of Yazd Research and Clinical Center for Infertility were used. Forty female mice were mated with male mice in serial days. When sperm plug was observed it was designated as gestational day (GD) (0). Pregnant mice were individually housed in plastic cages with ad libitum access to food and water in room with temperature between 22-26^o^C and 12-h/12-h light and dark cycle. MA dissolved with sterilized 0.9% saline, and MA or its vehicle were injected subcutaneously in a fixed volume of 0.1 ml/10g body weight. 

Pregnant mice were divided into four groups: first group received 10 mg/kg/day MA, second group received 5 mg/kg/day MA, and in third group saline (SAL) was administered from GD 6 to GD 14 between 09.00 am to 11.00 am, and fourth group was as control group without any injection. All groups weighted during this period. On GD 14, pregnant mice were dislocated, and then their fetuses were removed and weighted. 

**Chromosome preparation from fetal liver**

Fetal livers were removed and after washing once, cell suspension was provided with padding liver in a petri dish with the content of 1 pipetful prewarmed physiological serum from a9-inc Pasture pipet. The dish was tilted and the supernatant with suspended cells was transfered to a15-ml conical centrifuge tube and incubated with 200µl colchicine for 10 min at 37^o^C. Then the tube was centrifuged 5 min at 400 g (1300 rpm) and supernatant was removed and discarded. The pellet was resuspended by flicking bottom and 1 pipetful prewarmed 0.075 M KCL solution was added, and mixed gently, and let stand for 15 min in room temperature. 

Then it was centrifuged as before and supernatant was removed. Then the pellet was twice rinsed with 1 pipetful fixative solution without disturbing the pellet. 1/2 pipetful fresh fixative was added and let stand for 5-10 min until pellet was completely white. Then supernatant was removed and pellet was shaked, 1/2 pipetful of fresh fixative was added and centrifuged as before. In total three washes was repeated. Supernatant was removed and 1 ml fresh fixative was added and the slide was made and then stained by Gimsa staining method. 

**Fetal body size and head circumference measurement**

Crown-rump length of fixed fetuses and anterior-posterior and bilateral length of fetuses head was measured with caliper, then the head circumference was gained with the use of C=π {3(A+B)-[(3A+B) (A+3B)]1/2} formula. 

**Preparation of histological sections from fetal brain**

Fetuses were fixed on GD 14 in formalin for brain hematoxilin and eosine (H&E) staining and TUNEL assay. Fetal heads were decapitated and marked on bregma, then put in tissue processor. Heads were embedded in paraffin and then coronal serially sections with 5 µm thickness was provided from frontal region for H & E staining and bregma region, corresponding to the striatum for TUNEL assay, using Ziess, Axiphot, Germany, CCD Digital Camera, DXM 1200, Nikon, Japan. 

**H and E staining**

Brain sections (5 µm) were deparaffinized in xylen, followed by hydration in reduser ethanol serial and distillated water. Sections were staining by H&E then were dehydrated in ascendant ethanol and finally were left in xylen.

**Detection of cellular apoptosis by TUNEL assay**

After sections deparaffinized were in xylen and ethanol, in order to inhibit the androgen, proxidaze enzyme was used (3% H_2_O_2_) in methanol for 15 min. For increase membrane permability, proteinase K 20µg/ml of PBS was used and histological sections were incubated for 1h at 37^o^C. Then sections covered with TUNEL labeling solution, and incubated for 1h at 37^o^C. Then convertor POD for 40 min at 37^o^C and DAB substrate for 10-15 min used respectively. After each stage washing was performed with PBS. 

Finally, dehydration of sections was performed by ascendant ethanol and xylen. A serial section of striatum region of 5 mice in any group was used, and 1 section from any 5 section was studied with gaduate lense by 400 magnification then cells were counted and percentage of apoptotic cells were scored. 

**Statistical analysis**

Increase of maternal body weight during GD 6 to GD14, fetuse’ body weight and size, and head circumference in each group were compared using ANOVA. When the group interactions were significant, the Tukey HSD test was performed to evaluate post hoc comparisons among groups. t-test was used for comparison of number of apoptotic cells in fetal striatum between groups. P-value<0.05 was considered significant.

## Results

**Maternal and fetal body weights**

Only fetuses of 10 mg/kg MA group showed significantly lower body weight compared to control group (p<0.0001). Maternal body weight were not significantly difference between groups (p=0.173).

**Fetal body size and head circumference**

Measurements showed that head circumference was significantly decreased in first group compared to control group (p=0.020), but in other groups the difference was not significant. Length of fetal body size in experimental groups did not show significant difference compared to control group.

**Morphological abnormality**

**Figure 1:**
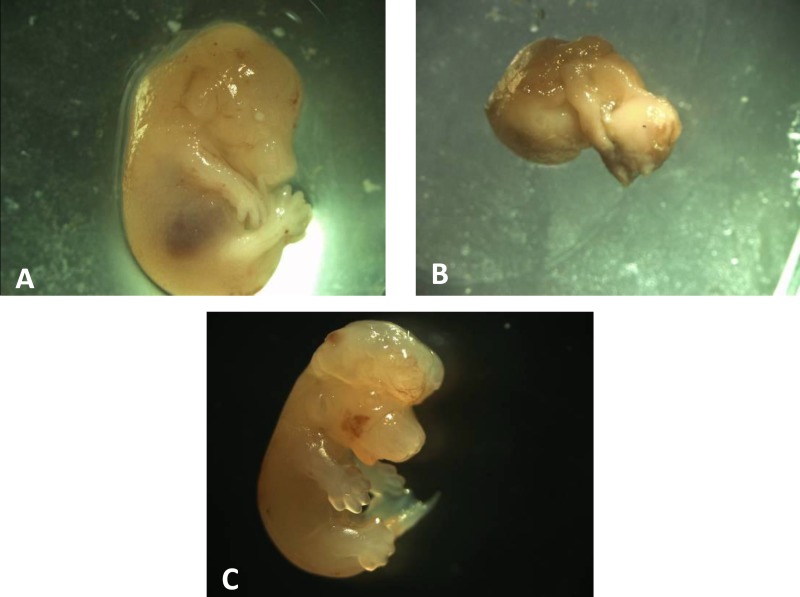
Morphological abnormality in methamphetamine (MA) fetuses. (A) normal fetus. (B) premature fetus of 10 mg/kg MA group. (C) exencephal fetus of 5mg/kg MA group.

Morphological study showed premature fetuses in 10 mg/kg MA group and exencephaly in fetus expose to 5 mg/kg MA (Figure 1B, C).There were no morphological abnormality in control and SAL groups (Figure 1A).

**Karyotyping analysis**

**Figure 2:**
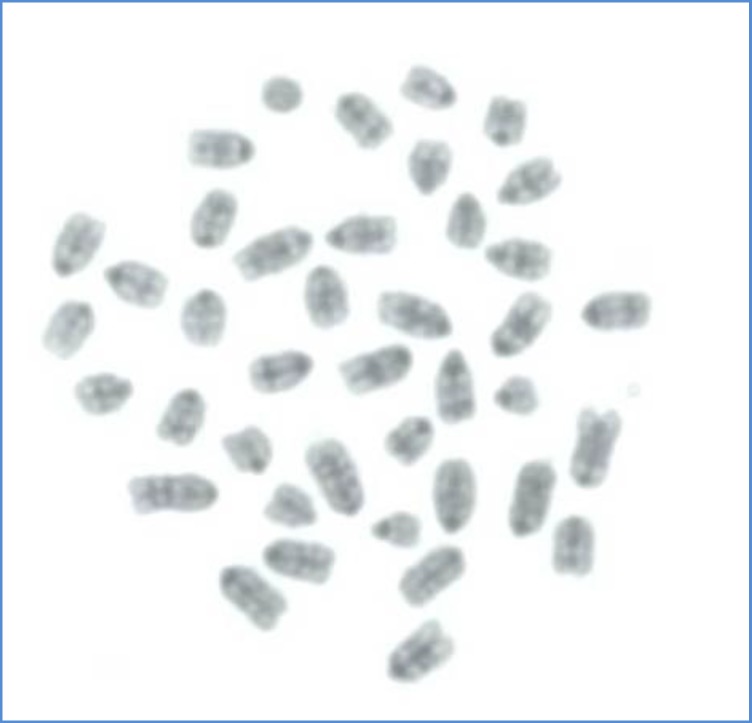
Karyotype of exencephal fetus from fetal liver on GD 14 (1000X)

There was not any numerical and structural chromosomal consitituren in fetus of all groups (Figure 2). 

**Subarachnoid hemorrhage**

**Figure 3.**
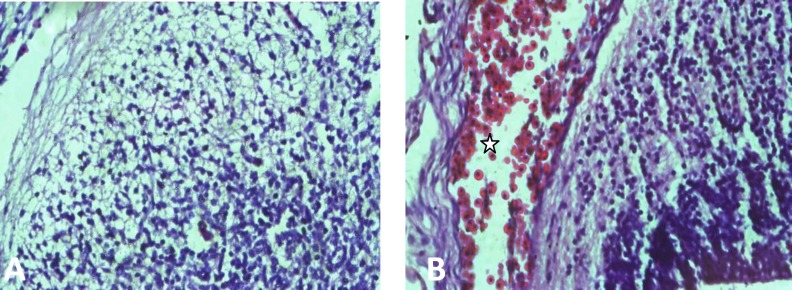
Subarachnoid hemorrhage in methamphetamine (MA) groups. (A) brain normal tissue. (B) subarachnoid hemorrhage in MA groups (*stare*). (400X)

Result of H&E staining showed subarachnoid hemorrhage in fetuses expose to 10 mg/kg and 5 mg/kg MA (Figure 3B). There were not histological changes in contral and SAL group (Figure 3A).

**Apoptosis**

**Figure 4:**
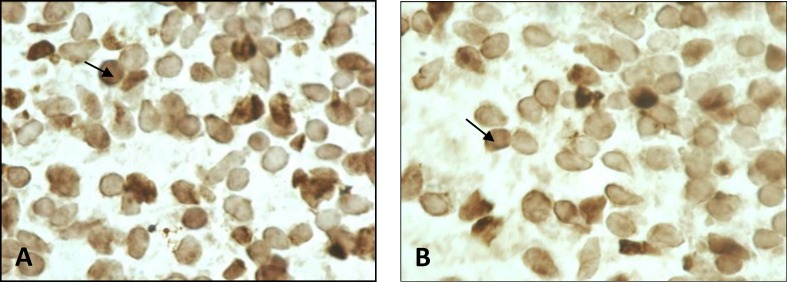
Apoptotic cell in striatum no difference in control and methamphetamine (MA) groups. (A) control group. (B) MA group. (TUNEL assay 1000X).

Number of apoptotic cells in striatum region in all groups was very close, and there were no significant difference between groups (Figure 4).

## Discussion

Rapid increase in illicit production and abuse of MA, popularity of MA between youth and abuse by women of childbearing age, encourage researcher to study various effects of exposure to MA (1). Many studies were performed on neurotoxic effects, and functional and behavioral impairments following MA exposure in adult human and animal (4, 14-17). But little studies were conducted on the teratogenic effects of MA, in particular that whether this malformation associated with chromosome abnormality. Therefore in this study, we examined effects of MA on fetal brain of mouse and possibility of existence chromosomal abnormality. Also, body weight of dams during injection, fetal body weight and length, and fetal brain circumference were compared between groups.

According to pharmacological properties of MA, which decreases appetite and increases the metabolism, a lower maternal body weight during MA injection was expected (20-22). But there were no significant difference between maternal body weight of MA exposure groups and control group. Although body weight of fetuses in 5 mg/kg MA group did not show significant difference, lower body weight in 10 mg/kg MA group was significantly lower compared to other groups (p<0.0001). Similar to human studies, that exposed to MA *in utero*, decrease of head circumference was observed in this study in 10 mg/kg MA group (p=0.02) (23). Therefore it is possible that treatment with higher dose can induced microcephaly.

Morphological observation showed a case of exencephaly in 5 mg/kg MA group and premature fetuses in 10 mg/kg MA group. Previous clinical and animal studies showed cleft palate, cardiac defects, exencephaly, limb reduction defects, eye abnormalities and skeletal malformations (24-27). In the present study we kept pregnant mouse in seperaty individual cage. Yamamoto and their colleagues in 2008 have shown that incidence of malformations was significantly lower in the groups kept individually than in crowded housing conditions. Some maternal physiological alterations caused by crowded environment such as stress-induced maternal hyperthermia and glucocorticoid secretion may be responsible for the potentiated teratogenicity of MA in aggregated groups (33).

In spite of induced malformations by MA, associated malformation with chromosomal defect is less known. The case of an infant with exencephaly has reported who was born to parents with a history of MA use during the pregnancy. Karyotype and subtelomeric FISH analysis on infant did not show any abnormality (34). But a case of holoprosencephaly and trisomy 13 has been described with maternal early gestational abuse of amphetamine (35). 

Our chromosomal evaluation that we performed on fetuses particularly exencephal fetus showed normal structure and number of chromosomes. H&E staining showed subarachnoid hemorrhage in MA groups, but no hemorrhage in SAL and control was observed. Approximately in all autopsy related to MA brain hemorrhage was reported, such as massive subarachnoid hemorrhage, hematoma in the corpus callosum, intracerebral hemorrhage and intraventicular hemorrhage. 

The explaination for brain hemorrhage is sympathetic effects of MA by the release of norepinephrine, significant increase of blood pressure and arterial spasm that eventually cause to cerebral vascular accident, such as subarachnoid hemorrhage. Also, Dixon and colleagues in 1989 reported brain hemorrhage in children prenatally exposed to MA (36). In chick embryos after amphetamine exposure caudal hematomas were found (37). Interestingly it have been shown that prenatal MA exposure in sheep induced elevation in blood pressure not only in dams but also in their fetuses (38).

We examine apoptotic cell death in fetal striatum. The striatum is a key component of the basal ganglia and the brain region with highest levels of dopamine, dopamine nerve endings, and dopamine receptors. There is evidence that it has a main role in cognitive, motor and limbic functions (39). Several reports have shown dopaminergic nerve terminal degeneration and apoptosis in adult mouse striatum by MA (4-13). These finding have shown the importance of striatum in such study. 

We examine induced apoptosis by MA in striatum on GD14. Studies indicate that onset of neuronogenesis in the mouse striatum starts from 12^th^ day of gestation and continue the first few days postnatally. The peak period of neuronogenesis is on the 14^th^ day of gestation (40). In the present study there was no significant difference in apoptotic cell in MA groups compared to control group. There are several plausible explanations for this finding. One possibility is enhanced oxidative DNA (8-oxoG) repair activity in fetal relative to adult organs (41). 

Also pregnant mice were individually kept which can be effective, because in this condition dam undergo lower hyperthermia than pregnant females which were housed in aggregated groups, and cell death in striatum was dependent on magnitude of hyperthermia (12). Moreover the study performed by Tokunaga and their colleagues in 2008 showed that administration of 5 or 10 mg/kg MA for 5 days in adult rat did not increase apoptotic cell in striatum, but single administration of 50 mg/kg MA can cause apoptosis in striatum (42). Therefore keeping the mice in aggregate groups and higher dose administration maybe induced apoptotic cell death in striatum.

## Conclusion

In conclusion, based on our observations, prenatal exposure to 5 and 10 mg/kg MA during organogenesis do not cause chromosomal defect and apoptotic cell death in striatum, but can cause teratogenic effects and subarachnoid hemorrhage in fetus.
